# Occlusion of LTP-Like Plasticity in Human Primary Motor Cortex by Action Observation

**DOI:** 10.1371/journal.pone.0038754

**Published:** 2012-06-06

**Authors:** Jean-François Lepage, Olivier Morin-Moncet, Vincent Beaulé, Louis de Beaumont, Francois Champoux, Hugo Théoret

**Affiliations:** 1 Department of Psychology, Université de Montréal, Montréal, Canada; 2 Hôpital Sainte-Justine Research Center, Montréal, Canada; 3 Douglas Mental Health University Hopsital, McGill University, Montréal, Canada; 4 Department of Audiology, Université de Montréal, Montréal, Canada; University of Bologna, Italy

## Abstract

Passive observation of motor actions induces cortical activity in the primary motor cortex (M1) of the onlooker, which could potentially contribute to motor learning. While recent studies report modulation of motor performance following action observation, the neurophysiological mechanism supporting these behavioral changes remains to be specifically defined. Here, we assessed whether the observation of a repetitive thumb movement – similarly to active motor practice – would inhibit subsequent long-term potentiation-like (LTP) plasticity induced by paired-associative stimulation (PAS). Before undergoing PAS, participants were asked to either 1) perform abductions of the right thumb as fast as possible; 2) passively observe someone else perform thumb abductions; or 3) passively observe a moving dot mimicking thumb movements. Motor evoked potentials (MEP) were used to assess cortical excitability before and after motor practice (or observation) and at two time points following PAS. Results show that, similarly to participants in the motor practice group, individuals observing repeated motor actions showed marked inhibition of PAS-induced LTP, while the “moving dot” group displayed the expected increase in MEP amplitude, despite differences in baseline excitability. Interestingly, LTP occlusion in the action-observation group was present even if no increase in cortical excitability or movement speed was observed following observation. These results suggest that mere observation of repeated hand actions is sufficient to induce LTP, despite the absence of motor learning.

## Introduction

It is well known that the acquisition of skilled motor performance depends heavily on practice. Typically, motor practice produces a fast initial gain in performance, which may be translated into long lasting behavioral changes under adequate conditions [1]. Animal experiments show that motor learning is associated with physiological changes within the primary motor cortex (M1), such as modification of motor maps [2] and strengthening of horizontal synaptic connections [3]. These changes in cortical representation and synaptic efficacy are thought to arise mainly from the early occurrence of long-term-potentiation (LTP) following motor practice [2]. Moreover, the disruption of LTP immediately following motor practice, either by practicing another task [4] or by external stimulation [5], prevents the expected behavioral gains from session to session, suggesting that M1 is one of the areas where learning initially occurs [5].

In humans, motor practice also produces noticeable changes at M1 paralleling what is reported in animal studies. These modifications include an increase in cortical excitability [6] and the modification of corticomotor representations of trained muscles [7], [8]. Brain stimulation makes it possible to study, in vivo, the physiological mechanisms hypothesized to be at the core of motor learning [9]. Paired-associative stimulation (PAS), where electric stimulation of sensory afferents is repeatedly coupled with transcranial magnetic stimulation (TMS) over the contralateral M1 at constant intervals, has been shown to be effective in inducing both LTP and LTD-like plastic changes at M1 [9], [10]. For example, it has been shown that electrical stimulation of the median nerve followed 25ms later by TMS over the contralateral representation of the abductor pollicis brevis (APB) muscle, when repeated over time at a frequency of 0.05 Hz, can increase MEP size for at least 60 minutes [9], [10] compared to pre-PAS values. Thus, this protocol aims at creating repeated, simultaneous inputs at the motor cortex level in accord with Hebbian plasticity principles. The duration, reversability, muscle specificity and NMDA-receptor dependence of the effect argue in favor of a PAS mechanism akin to actual LTP [11]. Furthermore, similarly to what is observed following motor learning in animals, experimentally-induced LTP is inhibited when immediately preceded by motor practice [11], [12] strongly suggesting that motor learning at M1 occurs, at least in part, through LTP [13].

It has been shown that observation of motor actions also induces activity in cortical motor areas [14]. Involvement of M1 during action observation has been particularly well described in humans with TMS. Using this technique, Fadiga and collaborators [14] showed that MEP amplitudes recorded from hand muscles were increased when participants passively observed hand movements. This facilitation during action observation has since been replicated numerous times [15]–[18] and appears to be muscle-dependent rather than direction dependent [19], temporally coupled with the observed action [20], causally linked to activity in premotor cortex [21] and dynamically modulated [22].

Numerous behavioral studies have shown that motor performance can be influenced positively by concomitant observation of simple movement [23]–[24]. Recent experiments have shown that action observation can induce the formation of a motor memory and impact *subsequent* motor performance. For example, Mattar and Gribble [25] showed that individuals observing a video depicting another person learning a motor task performed better than subjects who observed similar movements without learning. Positive consequences on motor performance were also seen by combining action observation and motor practice, whose combined effects were stronger than action observation [26]–[27] or physical practice [27] alone. At the neurophysiological level, Stefan and colleagues [28] showed that an extended observation period of thumb movements oriented in the opposite direction to that induced by TMS pulses could bias subsequent TMS-induced movement in favor of the observed direction.

Taken together, available data show that action observation can facilitate motor learning [25] and produce changes at M1 that closely resemble those elicited by actual practice of a repeated movement [28]. Since motor learning in humans is partly explained by fast LTP-like changes occurring at M1 [11], it can be hypothesized that passive observation of repeated movements would also lead to LTP-like effects. Here, a PAS protocol was used to determine whether observation of repeated thumb movements would lead to occlusion of LTP-like plasticity.

## Methods

### Ethics statement

All participants gave written informed consent, and the study protocol was approved by *the Comité d'éthique de la recherche des Sciences de la santé de l'Université de Montréal* and was conform to the Declaration of Helsinki.

### Participants

Thirty-six right-handed subjects (25 women; mean age 22±3 years, range 18 – 28 years) participated in the study. All of the participants reported being healthy, free of psychiatric or neurological antecedents, and were in compliance with inclusion/exclusion criteria regarding the safe use of TMS [29]. All participants gave written informed consent, and the study protocol was approved by *the Comité d'éthique de la recherche des Sciences de la santé de l'Université de Montréal* and was conform to the 1964 Declaration of Helsinki.

### Behavioral tasks

Prior to PAS, participants were randomly assigned to three experimental groups (n = 12) that each performed a different task. The *motor practice* condition was adapted from Muellbacher et al [30] and consisted of isolated thumb abduction movements soliciting the APB of the right hand performed as fast as possible (800 repetitions), paced by a brief sound signal at a rate of 0.5Hz. Subjects were seated comfortably in an upright position with fingers II-V of their right hand immobilized in a cast resting on a table in front of them, leaving the thumb free for movement in all directions. During the procedure, participants were asked to fixate a cross on a computer screen that was visible at all times. In the *action observation* condition, participants watched short videos depicting a series of thumb abductions peformed by a right male hand at 0.5Hz at an egocentric perspective and identical to those in the *motor practice* condition. To maintain attention on the task, participants were asked to report the number of thumb abductions in a given series of movements (25–35 per series; total 800). After each series, a cue appeared on the screen prompting the participant to verbally indicate the number of movements that were oberved. The next series started immediately after the participant*'*s response. In the *dot observation* condition, participants were asked to watch short videos depicting a moving dot of approximately 0.9° that closely matched biological movement in the *motor practice* and *action observation* conditions; the dot moved in a 90° semi-circular, counter-clockwise direction and back to its original position at a frequency of 0.5Hz (25–35 per series; total 800). Participants were also asked to count dot movements and report their number after each video sequence. Each condition lasted approximately 28 minutes and stimulus presentation was managed by Psyscope × running on a 17 inch- IMac computer (Apple, Cupertino, USA).

### Paired-associative stimulation

The PAS protocol was adopted from Ziemann et al [11]. It consisted of 200 electric stimulations of the right median nerve at the wrist paired with single TMS pulses delivered 25 ms later over the optimal region for eliciting MEPs in the right APB. Timing between electric and TMS pulses was controlled by a Quantum Composers 9514 pulse generator (Quantum Composers, Bozeman, USA). The rate of paired stimulation was 0.25 Hz. Electrical stimulation was applied through a bipolar electrode (cathode proximal) connected to a Digitimer DS7A (Digitimer Ltd, Hertfordshire, England) constant current stimulator, using square wave pulses (duration, 1 msec) at an intensity of three times the perceptual threshold. TMS pulses were delivered over the left M1 using a 80mm figure-of-eight coil connected to a Magstim 200 stimulator (The Magstim Company, Whitland, Wales, UK). The coil was angled 45° from the midline with the handle pointing backward. MEPs were recorded from electrodes placed over the contralateral APB muscle, and a circular ground electrode was placed over the participants*'* wrist. The electromyographic signal was amplified using a Powerlab 4/30 system (ADInstruments, Colorado Springs, USA), filtered with a band-pass 20–1000 Hz and digitized at a sampling rate of 4 kHz. MEPs were recorded using Scope v4.0 software (ADInstruments, Colorado Springs, USA) and stored offline for analysis. Prior to the experimental procedure, the stimulation site eliciting MEPs of maximal amplitude was determined. The intensity of stimulation was individually defined to elicit MEPs of approximately 1 mV in the APB at rest. To ensure stable coil positioning throughout the experiment, a Brainsight neuronavigating system (Rogue Research, Montréal, Canada) marking the site of stimulation was used. During the procedure, participants were asked to count the number of electrical stimulations to control for the known effects of attention on PAS-induced plasticity [31].

### Measurement of cortical excitability

Cortical excitability was assessed at four time points throughout the experimental session using a TMS intensity set to elicit MEPs of approximately 1 mV in amplitude in the resting APB before the experimental session. Twenty single TMS pulses were delivered with an interstimulus interval (ISI) of 6–7 seconds before (T1) and after (T2) the behavioral task, as well as 1 min (T3) and 10 minutes (T4) after PAS. Peak-to-peak amplitudes of the collected MEPs were measured and averaged at each time point for each participant.

### Motor learning

In a separate experiment, the behavioral effects of the three experimental conditions (*motor practice, action observation, dot observation*) were assessed following the method of Ziemann and collaborators [11]. Eighteen right-handed participants (8 women; mean age 26±5 years, range 21–37 years), none of whom took part in the TMS experiment, were randomly assigned to the three experimental groups (n = 6). Average speed (beginning to end of abduction) of fastest thumb abductions triggered by a sound was measured before and after the same behavioral tasks used in the TMS experiments. Participants observed either thumb abductions or dot movements at a rate of 0.5 Hz, or executed thumb abductions at a rate of 0.5 Hz, 800 times. Ten movements were performed before and after motor practice or observation at a rate of 0.1 Hz. A position sensor was attached to the tip of the participant*'*s hand and speed was measured using an Optotrak Certus motion capture system (Northern Digital Inc., Waterloo, Canada).

### Statistical analysis

Prior to analysis, data were inspected for the presence of multivariate outliers. Three cases (one in each group) had Mahalanobis distance values exceeding the critical value of D^2^ = 6.32 (df = 2; p<0.05) and were excluded from further analysis. The effects of motor practice and observation on peak acceleration and MEP size were assessed with two separate mixed ANOVAs with *Time* (T1, T2) as the within-subjects factor and *Group* (motor practice, action observation, dot observation) as the between-subjects factor. The effect of PAS on cortical excitability was tested using a mixed ANOVA with *Time* (T3, T4) as the within-subjects factor and *Group* (motor practice, action observation, passive control) as the between-subjects factor. MEP sizes for the T3 and T4 time points were normalized to the T2 value. When necessary, post-hoc analyses were conducted using Tukey HSD tests.

## Results

### MEP size

**Figure 1:**
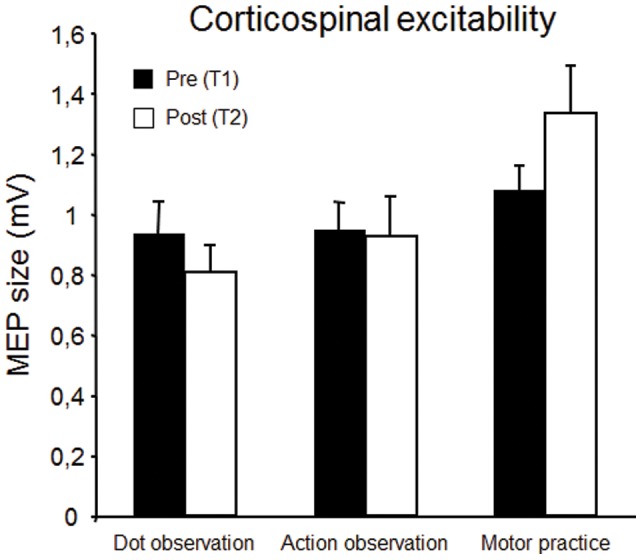
Corticospinal excitability before (black) and after (white) observation of moving dots, observation of thumb movements, or execution of thumb movements. Bars indicate standard error of the mean.

ANOVA with *Group* and *Time* (T1, T2) revealed a significant main effect of *Group* (F = 3.653; *p* = .038), no main effect of *Time* (F = 0.227; *p* = .637), and a trend towards significance for the interaction (F = 2.518; *p* = .097). This trend was manly driven by the presence of an increase in MEP size from T1 and T2 limited to the motor practice group (dot observation: 0.94±0.34mV → 0.81±0.30mV; action observation: 0.95±.30mV → 0.92±0.45mV; motor practice: 1.08±0.26mV → 1.34±0.51mV) (Figure 1, Table 1).

**Table 1:** Raw MEP values (mV). Data are presented as mean and SD.

Condition	T1	T2	T3	T4
Dot observation	0.94 (0.34)	0.81 (0.30)	1.20 (0.52)	1.21 (0.76)
Action observation	0.95 (0.30)	0.92 (0.45)	0.75 (0.37)	0.66 (0.30)
Motor practice	1.08 (0.26)	1.34 (0.52)	1.29 (0.81)	1.09 (0.49)

### Behavior

**Figure 2:**
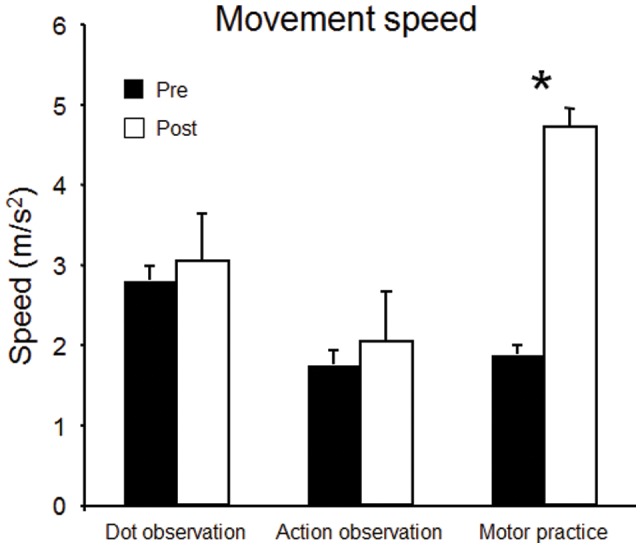
Speed of fastes thumb abduction before (T1, black) and after (T2, white) observation of moving dots, observation of thumb movements, or execution of thumb movements. Bars indicate standard error of the mean. * p<0.05.

ANOVA revealed a main effect of *Time* (F = 8.52; df = 1,15; *p* = 0.011), no main effect of *Group* (F = 3.34; df = 2,15; *p* = 0.063), and a significant interaction between factors (F = 4.98; df = 2,15; *p* = 0.022; Figure 2). Post hoc analysis showed that speed significantly increased only in the motor practice condition (*p* = 0.025). Motor practice: 1.87±0.62m/s^2^ → 4.71±2.06 m/s^2^; Action observation: 1.75±0.40m/s^2^ → 2.04±0.78m/s^2^; Dot observation: 2.80±0.59m/s^2^ → 3.04±1.98m/s^2^.

### PAS

**Figure 3:**
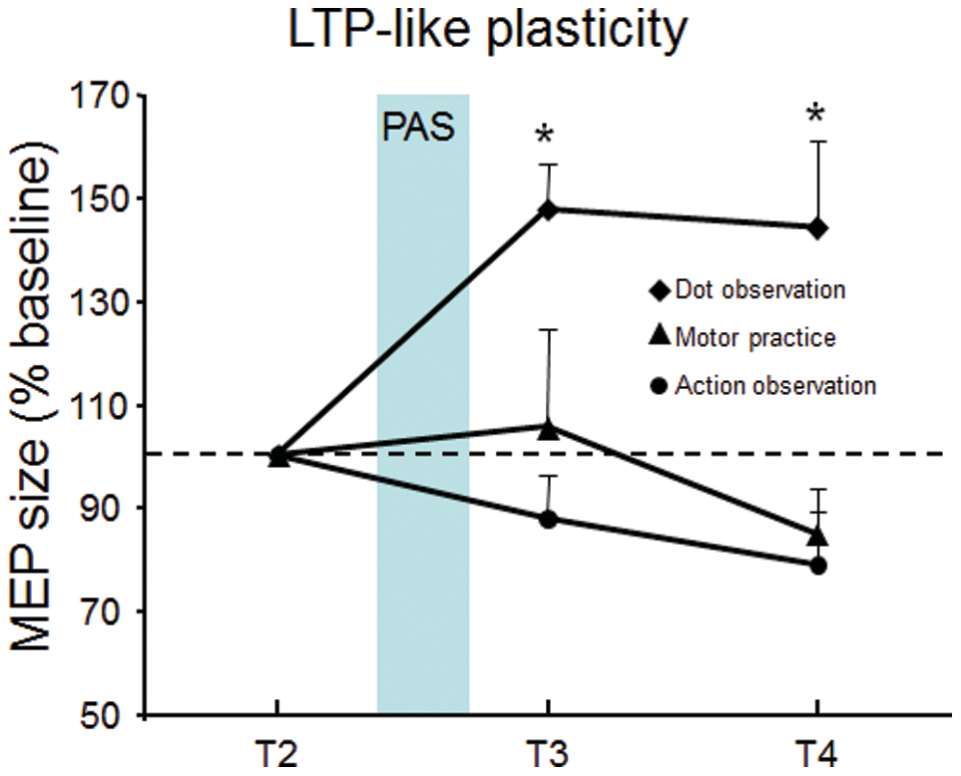
Interaction between observation of moving dots, observation of thumb movements and execution of thumb movements with PAS. Data are expressed as a percent change from pre-PAS values (T2) immediately (T3) and ten minutes (T4) after the end of PAS. Bars indicate standard error of the mean. * p<0.01.

As commonly used in similar protocols [11], a mixed ANOVA was conducted with MEP amplitudes following PAS intervention normalized to the time point immediately preceding it (T3/T2, T4/T2). This revealed a significant main effect of *Group* (F = 8.303; df = 2,30; *p* = 0.001), no main effect of *Time* (F = 3.170; df = 1,30; *p* = .085) and no interaction between factors (F = 0.666; df = 2,30; *p* = .521). Post-hoc analysis revealed that PAS induced greater increases in MEP size in the *dot observation* group than in both the *action observation* (p  = .002) and *motor practice* (p  = .006) groups, with no significant difference between *action observation* and *motor practice* (p  = .938) (Figure 3, Table 1).

## Discussion

The present results show that observation of physical movement is sufficient in preventing the subsequent occurrence of LTP-like plasticity in M1, similarly to what is observed after physical practice. Additionally, the reduction in LTP-like plasticity following action observation can occur despite the absence of significant increases in movement speed and corticospinal excitability. Group differences in corticospinal excitability at baseline and lack of a significant increase in excitability between T1 and T2 in the motor practice group limit the conclusions that can be drawn from the present data. Nevertheless, the results strongly suggest that both action observation and execution induce similar plasticity modification in the motor cortex as revealed by PAS.

Pairing repeated electrical stimulation of the median nerve with magnetic stimulation of the cortical representation of the APB muscle increases cortical excitability if both inputs converge into primary motor cortex at approximately the same time [9]. This effect is believed to reflect LTP-like plasticity, partly because of its dependence on NMDA receptors [32]. In the present study, this effect was replicated in the control condition, where participants observed moving dots prior to the PAS protocol, such that corticospinal excitability was increased for at least 10 minutes after the end of paired stimulation. Furthermore, LTP-like plasticity failed to occur when PAS was delivered after 30 minutes of thumb abduction movements, also replicating previous findings [11]–[13]. Contrary to similar studies, however, physical practice did not lead to a significant increase in corticospinal excitability when MEP sizes were compared before and after motor practice (e.g [11], [30]). Despite the lack of a significant increase in corticospinal excitability, some lines of evidence suggest that motor learning could have occured in M1 during motor practice: *i)* motor practice led to a 24 % increase in corticospinal excitability whereas the dot observation and movement observation conditions both led to small *decreases* in M1 excitability; *ii)* there was a statistical trend towards significance for the interaction between the Time and Group variables, driven by the increase in MEP size from T1 to T2 in the motor practice group; *iii)* 8 out of 11 participants showed increased corticospinal excitability following motor practice; *iv)* behavioral data showed significantly increased movement speed after motor practice. Nevertheless, it remains that motor training did not produce a statistically significant effect on MEP size, leading us to conclude that motor learning occurred in the motor practice group (behaviorally) despite any significant change in corticospinal excitability, contrary to previous findings [11], [12].

The main finding of the present study is that 30 minutes of repeated thumb movement *observation* significantly reduced the effects of PAS, similarly to what is seen after physical practice. It has been suggested that occlusion of LTP-like plasticity in human motor cortex by motor learning can be explained by saturation of the synaptic modification range [11], [13], a notion that is consistent with animal experiments [33]. With regards to the present findings, this would suggest that mere observation of repeated actions induces LTP-like plasticity in primary motor cortex, resulting in an increased threshold for subsequent induction of LTP. Interestingly, however, occlusion of LTP-like plasticity during action observation occurred despite the absence of motor learning and motor learning-induced changes in corticospinal excitability. In fact, observation of thumb movements did not increase movement speed compared to baseline levels and TMS-induced MEPs at the APB were of similar size before and after action observation. In a similar design, Stefan and collaborators [28] measured the direction and speed of TMS-evoked thumb movements before and after 30 minutes of passive observation of thumb movements in a direction opposite to that normally elicited by TMS. It was found that after observation, both acceleration and direction of TMS-evoked thumb movements were modified according to the direction of the previously observed movement sequence. These modifications occurred despite the fact that corticospinal excitability of the agonist muscle did not increase following thumb observation. Rather, movement observation enhanced corticospinal excitability only when the ratio of the agonist over the antagonist muscle was compared pre- and post-observation. TMS-induced muscle activity in the antagonist muscle was not recorded in the present study, leaving open the possibility that corticospinal excitability modulation could have occurred in the action observation condition.

The absence of learning effects in the observation condition suggests that a different mechanism may explain subsequent LTP occlusion. Learning by observation and the formation of “motor memories” at M1 induced by action observation have been suggested to involve, at least partially, the human mirror neuron system (hMNS; [25], [28]). Numerous TMS studies have shown that passive observation of transitive and intransitive movements is associated with increases in corticospinal excitability that are highly muscle-specific, that strictly follow the dynamic nature of the movement, and can simulate future actions in the absence of actual movement (e.g [14], [17], [22], [34]). One possible modulating source of M1 activity during action observation is cortico-cortical connections originating in ventral premotor cortex (PMv; [14]). The PMv is a central component of the hMNS [35] and is richly connected with M1 [36], [37]. Avenanti and collaborators [21], [38] have shown that disrupting PMv with repetitive TMS significantly reduces the facilitation in corticospinal excitability that occurs during action observation. Conversely, it was found that disruption of M1 activity did not modulate the facilitatory effects associated with action observation, suggesting that M1 does not directly participate in motor mirror responses [21]. This suggestion was recently supported by two-coil, paired pulse data showing increased MPv-M1 connectivity during observation of grasping movements [39]. Furthermore, it has recently been shown that PMv directly contributes to the facilitatory effects of action observation on use-dependent plasticity. In a disruption study, Cantarero and collaborators [40] reported that 1 Hz rTMS over PMv abolished the behavioral gains resulting from the addition of action observation to physical practice. Taken together, these data suggest that mirror activity in PMv created by repeated exposure to thumb movements may modulate M1 excitability in such a way that the threshold for subsequent LTP induction in increased.

Alternative explanations for the present results also need to be addressed. For example, it has been shown that rTMS protocols that do not induce plasticity, such as 0.1 Hz rTMS, can occlude subsequent induction of both LTD- and LTP-like plasticity by PAS [41]. This is similar to the present data showing occlusion of LTP-like plasticity following action observation despite the lack of increased corticospinal excitability. As suggested by Delvendahl and collaborators [41], this biderectional occlusion effect requires an alternative explanation to the sliding threshold described earlier, perhaps in the form of gating. Since observation of thumb movements in the present study did not create plasticity in primary motor cortex in the form of increased excitability, it may be argued that similarly to 0.1 Hz rTMS, action observation increased intracortical inhibition and prevented subsequent LTP-like plasticity. Another mechanism that may be involved in the occlusion of LTP-like plasticity by action observation is changes in spinal excitability. Meunier and collaborators [42] have shown that PAS induces changes in H-reflex recruitment curves, suggesting a possible interaction between motor practice, movement observation and PAS-induced plasticity at the spinal cord level. Further studies using paired-pulse and H-reflex protocols are needed to directly test these hypotheses.

An important limitation of the present study is the fact that baseline differences in corticospinal excitability were present between the motor practice and action observation groups. This group difference was not due to the presence of statistical outliers, as they were removed prior to analysis based on Mahalanobis distance values, suggesting that both groups differed in baseline excitability before training began. Therefore, it could be argued that baseline differences may have modified the between-group response to both motor learning-dependent MEP changes and the subsequent response to PAS. With regards to possible counfound associated with groups entering the PAS protocol at different levels of corticospinal excitability, Ziemann and collaborators (2004) have shown that correcting MEP amplitude before PAS to baseline levels (1mV peak-to-peak) has no effect on the M1 response to PAS. This does not preclude, however, the possibility that baseline differences at T1 may somehow have played a part in group responses to PAS observed in the present study. As such, one can only safely assume that plasticity in primary motor cortex is modified in a similar way after motor practice or action observation, despite differences in baseline levels. Indeed, the raw data presented in Table 1 clearly show that the action observation and motor practice groups react in a similar manner: the PAS-induced increase from T2 to T3 (after PAS) does not occur, event though the motor practice group started from a higher baseline. Crucially, there is a clear difference between the action observation group (no PAS effect) and the dot observation group (PAS effect), whose baseline values were very similar.

The potential utility of action observation as a rehabilitative tool has received much attention from clinicians and neuroscientists in the past few years [43], and there are now some reports of beneficial effects of action observation on motor function used in conjunction with motor practice in healthy [44] and physically impaired individuals [45]. The present results provide some indication as to the underlying neurophysiological mechanism related to these behavioral gains, and suggest that an extended period of action observation may be sufficient to induce LTP in the primary motor cortex. Future studies looking at motor learning through action-observation should use larger samples to investigate its potency to induce long-lasting plastic changes within M1, such as modifications of motor maps and potentially, the existence of behavioral gains after consolidation of the newly created motor memory.
